# Supplementary material for: Pan-cancer analysis demonstrates that integrating polygenic risk scores with modifiable risk factors improves risk prediction
Source: Nat Commun. 2020 Nov 27;11:6084. doi: 10.1038/s41467-020-19600-4 (PMC7695829; doi:10.1038/s41467-020-19600-4)
Supplement: Supplementary file 1 — Supplementary Information [file 41467_2020_19600_MOESM1_ESM.pdf]

Supplementary Table 1: Characteristics of the UK Biobank study population, restricted to participants of predominately European ancestry, stratified by incident cancer status.

Baseline Characteristic	Status at the end of follow-up			
	Cancer-free (n=390,998)		Incident Cancer (n=22,755)	
	N	(%)	N	(%)
Age at assessment (years)				
Mean (SD)	56.53	(8.02)	60.19	(6.88)
Sex				
Females	211513	(54.10)	11080	(48.69)
Males	179485	(45.90)	11675	(51.31)
Smoking status				
Never	211936	(54.20)	10610	(46.63)
Ever	177685	(45.44)	12049	(52.95)
Former	137493	(35.16)	9174	(40.32)
Current	40192	(10.28)	2875	(12.63)
Pack-years: Mean (SD)	23.17	(15.61)	27.70	(19.19)
Unknown	1377	(0.35)	96	(0.42)
Body-mass index (kg/m^2)				
Normal: 18.5 ≤BMI<25	127817	(32.69)	6614	(29.07)
Underweight: <18.5	1984	(0.51)	96	(0.42)
Overweight: 25 ≤BMI<30	165859	(42.42)	10055	(44.19)
Obese: BMI≥30	94070	(24.06)	5914	(25.99)
Unknown	1268	(0.32)	76	(0.33)
Frequency of alcohol consumption				
Never	25864	(6.61)	1651	(7.26)
Special occasions	41557	(10.63)	2495	(10.96)
1-3 times per month	43627	(11.16)	2312	(10.16)
1-2 times per week	102999	(26.34)	5753	(25.28)
3-4 times per week	94177	(24.09)	5225	(22.96)
Daily	82482	(21.10)	5304	(23.31)
Unknown	292	(0.07)	1651	(7.26)
Ever diagnosed with hypertension?				
Yes	92541	(23.67)	6772	(29.76)
Any first-degree relative diagnosed with cancer[1]?				
Yes	139833	(35.76)	9195	(40.41)
Ever had screening for breast, prostate, or colorectal cancer?				
Yes	258249	(66.05)	16908	(74.30)
Deaths occurring during follow-up				
Death from any cause	5982	(1.53)	4878	(21.44)
Death due to cancer	2103	(0.54)	4696	(20.64)

1. Based on self-reported cancers of the breast, prostate, lung, or bowel in non-adopted parents and siblings

Supplementary Table 2: Risk factors in addition to age and sex (if applicable), such as environmental exposures, lifestyle factors, and family history, that were included in the most comprehensive model for each cancer. Risk factors were selected based on literature review and availability in the UK Biobank cohort.

Cancer Site	Risk Factors	Model Specification Notes
Prostate	Family history of prostate cancer	
Testis	-	
Breast	Family history of breast cancer, parity (≥1 live birth vs. none), age at menarche (years), menopausal status (pre-menopausal vs. post-menopausal vs. unknown or hysterectomy), ever used hormone replacement therapy (HRT), duration of oral contraceptive use (never used (0) vs. <20 years vs. ≥20 years), body mass index (BMI), weekly alcohol intake (grams)[1]	Interactions: (BMI)*(Menopausal status)
Endometrium	Family history of cancer, parity (≥1 live birth vs. none), age at menarche (years), menopausal status (pre-menopausal vs. post-menopausal vs. unknown or hysterectomy), ever used HRT, duration of oral contraceptive use (never used (0) vs. <20 years vs. ≥20 years), BMI	
Ovary	Family history of breast cancer, parity (≥1 live birth vs. none), menopausal status (pre-menopausal vs. post-menopausal vs. unknown or hysterectomy), ever used HRT, duration of oral contraceptive use (never used (0) vs. <20 years vs. ≥20 years), BMI	Interactions: (BMI)*(Menopausal status)
Cervix	Parity (≥1 live birth vs. none), duration of oral contraceptive use (never used (0) vs. <20 years vs. ≥20 years), cigarette pack-years	
Colorectum	Family history of bowel cancer, waist to hip ratio (WHR), cigarette pack-years, frequency of processed meat intake (<1 per week vs. ≥1 per week), moderate and/or strenuous physical activity (days per week), weekly alcohol intake (grams)	
Melanoma	Frequency of UV protection use (always vs. most times vs. sometimes vs. never out in the sun vs. never), time spent outside in the summer (hours per day), ease of tanning (very easily vs. moderate vs. mild vs. mostly burn)	
Lung	Family history of lung cancer, cigarettes per day (0 for never smokers), years of smoking (0 for never smokers), smoking status (never vs. former vs. current), $PM_{2.5}$ level in 2010 (micro-g/m^3)	Interactions: (Former smoker)* (cigarettes/day) (Former smoker)*(years of smoking)
Never-smokers	Family history of lung cancer, $PM_{2.5}$ level in 2010 (micro-g/m^3)	
Smokers	Family history of lung cancer, cigarettes per day, years of smoking, smoking status (former vs. current), years since quitting smoking (0 for current smokers), $PM_{2.5}$ level in 2010 (micro-g/m^3)	Interactions: (Smoking status)*(cigarettes/day) (Smoking status)*(years smoking)
NHL	-	
Bladder	BMI, smoking status (never vs. former vs. current), cigarette pack-years	
Kidney	BMI, smoking status (never vs. former vs. current), cigarette pack-years, ever diagnosed with hypertension	
Pancreas	BMI, smoking status (never vs. former vs. current), cigarette pack-years, family history of cancer (prostate, breast, lung or bowel)	
Oral cavity/pharynx	Smoking status (never vs. former vs. current), cigarette pack-years, weekly alcohol intake (grams)	
Lymphocytic leukemia	-	
Thyroid	BMI categories (BMI <25 vs. 25 ≤BMI <30, BMI≥30)	

1. Weekly alcohol intake was derived by summing up the total number of drinks per week across different types of alcoholic beverages (beer, wine, spirits) and converting to units of alcohol based on values from UK Composition of foods integrated dataset: https://www.gov.uk/government/publications/composition-of-foods-integrated-dataset-cofid

Supplementary Table 3: Hazard ratios (HR) and corresponding p-values for each cancer risk factor estimated using a cause-specific Cox regression model accounting for death as a competing risk.

Cancer Site and Risk Factors	HR[1]	(95% CI)	P-value
Prostate			
Family history of prostate cancer	1.84	(1.69 - 2.00)	9.1×10^{-46}
Breast			
Family history of breast cancer	1.56	(1.44 - 1.69)	3.0×10^{-29}
Parity (≥1 live birth)	0.91	(0.84 - 0.98)	0.010
Age at menarche (per 1 year)	0.99	(0.97 - 1.00)	0.14
BMI (per 1-unit increase)	0.99	(0.98 - 1.00)	0.11
Menopausal status: pre-menopausal	1.00		
Menopausal status: post-menopausal	0.36	(0.24 - 0.53)	1.8×10^{-7}
Menopausal status: unknown/hysterectomy	0.32	(0.18 - 0.55)	5.0×10^{-5}
Ever used hormone replacement therapy (HRT)	1.09	(1.02 - 1.17)	7.0×10^{-3}
Oral contraceptive use: 0 (never used)	1.00		
Oral contraceptive use: <20 years	1.00	(0.93 - 1.08)	0.94
Oral contraceptive use: ≥20 years	1.10	(0.98 - 1.23)	0.12
Alcohol intake[2] (70 g/week)	1.04	(1.02 - 1.05)	2.3×10^{-5}
BMI * menopausal status (post-menopausal)			2.0×10^{-5}
BMI * menopausal status (unknown/hysterectomy)			7.6×10^{-4}
Endometrium			
Family history of cancer	1.11	(0.95 - 1.30)	0.20
Parity (≥1 live birth)	0.64	(0.53 - 0.77)	3.3×10^{-6}
Age at menarche (per 1-year increase)	0.92	(0.88 - 0.97)	1.8×10^{-3}
BMI (per 1-unit increase)	1.09	(1.08 - 1.10)	1.6×10^{-49}
Menopausal status: pre-menopausal	1.00		
Menopausal status: post-menopausal	1.01	(0.73 - 1.39)	0.97
Menopausal status: unknown/hysterectomy	0.02	(0.00 - 0.08)	6.1×10^{-8}
Ever used HRT	0.84	(0.71 - 0.99)	0.041
Oral contraceptive use: 0 (never used)	1.00		
Oral contraceptive use: <20 years	0.83	(0.70 - 1.00)	0.051
Oral contraceptive use: ≥20 years	0.36	(0.24 - 0.56)	4.9×10^{-6}
Ovary			
Family history of breast cancer	1.30	(1.00 - 1.70)	0.051
Parity (≥1 live birth)	0.72	(0.57 - 0.91)	6.2×10^{-3}
BMI (per 1-unit increase)	1.04	(1.00 - 1.08)	0.036
Menopausal status: post-menopausal	3.19	(0.88 - 11.56)	0.08
Menopausal status: unknown/hysterectomy	1.07	(0.13 - 8.52)	0.95
Ever used HRT	0.97	(0.79 - 1.19)	0.79
Duration of oral contraceptive use: <20 years	0.82	(0.66 - 1.03)	0.09
Duration of oral contraceptive use: ≥20 years	0.57	(0.37 - 0.88)	0.012
BMI * menopausal status (post-menopausal)			0.023
BMI * menopausal status (unknown/hysterectomy)			0.45
Cervix			
Parity: ≥1 live birth	1.81	(1.30 - 2.53)	4.9×10^{-4}

Oral contraceptive use: 0 (never used)			
Oral contraceptive use: <20 years	0.84	(0.58 - 1.21)	0.34
Oral contraceptive use: ≥20 years	1.08	(0.69 - 1.69)	0.74
Cigarette pack-years (per 10 pack-years)	1.15	(1.05 - 1.25)	1.8×10^{-3}
Colon/rectum			
Family history of bowel cancer	1.26	(1.14 - 1.40)	1.2×10^{-5}
Waist to hip ratio (per 10% increase)	1.17	(1.11 - 1.24)	2.2×10^{-8}
Cigarette pack-years (per 10 pack-years)	1.04	(1.02 - 1.06)	2.1×10^{-4}
Processed meat intake: never	1.00		
Processed meat intake: < once a week	0.99	(0.98 - 1.00)	0.20
Processed meat intake: ≥ once a week	1.08	(0.92 - 1.28)	0.34
Physical activity: strenuous or moderate (days/week)	1.15	(0.98 - 1.35)	0.09
Alcohol intake[2] (70 g/week)	1.04	(1.03 - 1.05)	5.9×10^{-9}
Melanoma			
Apply UV protection: never	1.00		
Apply UV protection: sometimes	1.37	(1.10 - 1.69)	4.1×10^{-3}
Apply UV protection: most times	1.82	(1.48 - 2.25)	2.1×10^{-8}
Apply UV protection: always	1.68	(1.34 - 2.09)	5.3×10^{-6}
Apply UV protection: never in the sun	1.06	(0.46 - 2.42)	0.89
Time outdoors in the summer (hours per day)	1.03	(1.01 - 1.05)	7.3×10^{-3}
Ease of tanning: get very tan	1.00		
Ease of tanning: moderate	1.33	(1.16 - 1.53)	6.4×10^{-5}
Ease of tanning: mild	1.60	(1.37 - 1.86)	1.9×10^{-9}
Ease of tanning: mostly burn	1.61	(1.37 - 1.89)	4.0×10^{-9}
Lung			
Family history of lung cancer	1.61	(1.43 - 1.81)	7.4×10^{-15}
$PM_{2.5}$ in 2010 (per 1 micro-g/m^3)	1.10	(1.05 - 1.15)	1.9×10^{-5}
Cigarettes per day	1.00	(0.99 - 1.00)	0.52
Years of smoking	1.07	(1.06 - 1.09)	9.0×10^{-23}
Smoking status: never	1.00		
Smoking status: former	0.34	(0.25 - 0.46)	6.9×10^{-13}
Smoking status: current	0.84	(0.44 - 1.61)	0.60
Smoking status (former) * cigarettes per day			2.2×10^{-8}
Smoking status (former) * years of smoking			0.40
Lung (Never smokers)			
Family history of lung cancer	0.91	(0.61 - 1.38)	0.67
$PM_{2.5}$ in 2010 (per 1 micro-g/m^3)	0.93	(0.81 - 1.07)	0.32
Lung (Current or former smokers)			
Family history of lung cancer	1.71	(1.51 - 1.94)	3.8×10^{-17}
$PM_{2.5}$ in 2010 (per 1 micro-g/m^3)	1.12	(1.07 - 1.18)	1.2×10^{-6}
Cigarettes per day	1.02	(1.02 - 1.03)	1.2×10^{-13}
Years of smoking	1.07	(1.05 - 1.10)	1.4×10^{-8}
Smoking status: former	1.00		
Smoking status: current	2.12	(0.92 - 4.89)	0.08
Years since quitting (per 1 year)	1.01	(0.98 - 1.04)	0.44
Smoking status (current) * cigarettes per day			2.4×10^{-8}

Smoking status (current) * years of smoking			0.51
Bladder			
Cigarette pack-years (per 10 pack-years)	1.07	(1.03 - 1.11)	3.4×10^{-4}
Smoking status: never	1.00		
Smoking status: former	1.52	(1.26 - 1.82)	8.0×10^{-6}
Smoking status: current	2.07	(1.62 - 2.64)	5.7×10^{-9}
BMI (per 1-unit increase)	1.01	(0.99 - 1.02)	0.45
Kidney			
BMI (per 1-unit increase)	1.04	(1.02 - 1.05)	1.7×10^{-6}
Smoking status: never	1.00		
Smoking status: former	1.07	(0.87 - 1.32)	0.52
Smoking status: current	1.36	(1.02 - 1.83)	0.039
Cigarette pack-years (per 10 pack-years)	1.07	(1.02 - 1.12)	7.9×10^{-3}
Diagnosed with hypertension	1.69	(1.44 - 1.98)	2.1×10^{-10}
Pancreas			
Family history of cancer (prostate, breast, lung, bowel)	1.40	(1.17 - 1.67)	1.9×10^{-4}
BMI (per 1-unit increase)	1.03	(1.01 - 1.05)	8.4×10^{-4}
Cigarette pack-years (per 10 pack-years)	1.04	(0.97 - 1.10)	0.27
Smoking status: never	1.00		
Smoking status: former	1.08	(0.84 - 1.39)	0.56
Smoking status: current	2.04	(1.46 - 2.84)	2.8×10^{-5}
Oral cavity/pharynx			
Alcohol intake[2] (70 g/week)	1.05	(1.04 - 1.07)	3.0×10^{-10}
Cigarettes per day	1.01	(1.00 - 1.02)	8.2×10^{-3}
Years of smoking	1.02	(1.01 - 1.04)	2.0×10^{-4}
Smoking status: never	1.00		
Smoking status: former	0.58	(0.37 - 0.91)	0.050
Smoking status: current	1.09	(0.60 - 1.96)	0.43
Thyroid			
BMI: <25	1.00		
BMI: 25 to <30	1.43	(1.01 - 2.02)	0.045
BMI: 30 to <35	1.59	(1.05 - 2.41)	0.028
BMI: ≥35	1.15	(0.61 - 2.15)	0.67

1. In addition to the listed risk factors all Cox regression models were adjusted for age and sex (if applicable)
2. Weekly alcohol intake was derived by summing up the total number of drinks per week across different types of alcoholic beverages (beer, wine, spirits) and converting to units of alcohol based on values from UK Composition of foods integrated dataset: https://www.gov.uk/government/publications/composition-of-foods-integrated-dataset-cofid

Supplementary Table 4: Hazard ratios (HR) per one standard deviation (SD) increase in the standardized polygenic risk score (PRS) and corresponding p-values were estimated using cause-specific Cox proportional hazards models, accounting for mortality as a competing risk. Results comparing three types of weighting approaches for combining individual risk variants in the PRS are presented: standard weights based on log odds ratios (PRS_{β}), unweighted sum of risk alleles (PRS_{unw}), and inverse variance (IV) weights (PRS_{IV}).

Cancer Site	Cases	PRS Description		HR[1]	(95% CI)	P-value	C index[2]	(C SE)	AUC[3]
		Variants	Weights						
Prostate	4740	161	PRS_{β}	1.39	(1.35-1.43)	2.0×10^{-105}	0.738	(0.004)	0.740
			PRS_{unw}	1.66	(1.62-1.71)	3.4×10^{-266}	0.759	(0.004)	0.761
			PRS_{IV}	1.77	(1.72-1.82)	4.3×10^{-336}	0.768	(0.004)	0.769
Testis	52	52	PRS_{β}	2.18	(1.66-2.87)	2.3×10^{-8}	0.749	(0.034)	0.783
			PRS_{unw}	1.96	(1.49-2.58)	1.4×10^{-6}	0.745	(0.035)	0.769
			PRS_{IV}	2.26	(1.71-2.99)	1.0×10^{-8}	0.766	(0.033)	0.787
Breast	4760	162	PRS_{β}	1.52	(1.47-1.56)	3.2×10^{-183}	0.632	(0.005)	0.637
			PRS_{unw}	1.42	(1.38-1.46)	2.1×10^{-129}	0.618	(0.005)	0.623
			PRS_{IV}	1.52	(1.47-1.56)	1.2×10^{-180}	0.635	(0.004)	0.637
Endometrium	643	9	PRS_{β}	1.19	(1.10-1.29)	1.1×10^{-5}	0.749	(0.011)	0.755
			PRS_{unw}	1.18	(1.09-1.28)	2.4×10^{-5}	0.749	(0.011)	0.754
			PRS_{IV}	1.18	(1.09-1.27)	3.5×10^{-5}	0.749	(0.011)	0.754
Ovary	445	36	PRS_{β}	1.13	(1.04-1.24)	6.2×10^{-3}	0.655	(0.015)	0.656
			PRS_{unw}	1.18	(1.07-1.29)	5.8×10^{-4}	0.652	(0.016)	0.658
			PRS_{IV}	1.20	(1.10-1.32)	9.0×10^{-5}	0.654	(0.015)	0.660
Cervix	282	10	PRS_{β}	1.22	(1.09-1.37)	7.3×10^{-4}	0.750	(0.017)	0.745
			PRS_{unw}	1.20	(1.07-1.35)	1.5×10^{-3}	0.749	(0.017)	0.745
			PRS_{IV}	1.21	(1.07-1.35)	1.5×10^{-3}	0.749	(0.017)	0.745
Colon/rectum	2725	103	PRS_{β}	1.32	(1.27-1.37)	5.5×10^{-50}	0.704	(0.006)	0.704
			PRS_{unw}	1.46	(1.41-1.52)	9.2×10^{-87}	0.714	(0.006)	0.714
			PRS_{IV}	1.48	(1.43-1.54)	1.8×10^{-94}	0.716	(0.006)	0.716
Melanoma	1805	24	PRS_{β}	1.43	(1.36-1.49)	5.7×10^{-51}	0.663	(0.008)	0.652
			PRS_{unw}	1.43	(1.36-1.49)	1.2×10^{-50}	0.662	(0.008)	0.652
			PRS_{IV}	1.44	(1.37-1.50)	2.4×10^{-53}	0.664	(0.008)	0.654
Lung	1541	109	PRS_{β}	1.16	(1.11-1.22)	1.5×10^{-9}	0.849	(0.006)	0.846
			PRS_{unw}	1.15	(1.09-1.20)	1.5×10^{-8}	0.849	(0.006)	0.846
			PRS_{IV}	1.17	(1.12-1.23)	1.2×10^{-10}	0.849	(0.006)	0.846
NHL	970	19	PRS_{β}	1.16	(1.09-1.24)	1.0×10^{-6}	0.676	(0.010)	0.677
			PRS_{unw}	1.18	(1.11-1.25)	2.9×10^{-7}	0.675	(0.010)	0.678
			PRS_{IV}	1.15	(1.08-1.22)	1.1×10^{-5}	0.674	(0.010)	0.677
Bladder	890	15	PRS_{β}	1.28	(1.20-1.37)	2.1×10^{-13}	0.813	(0.008)	0.803
			PRS_{unw}	1.30	(1.21-1.39)	7.6×10^{-15}	0.814	(0.008)	0.803
			PRS_{IV}	1.30	(1.22-1.39)	1.5×10^{-15}	0.814	(0.008)	0.804
Kidney	612	19	PRS_{β}	1.16	(1.08-1.26)	1.0×10^{-4}	0.724	(0.011)	0.722
			PRS_{unw}	1.13	(1.05-1.22)	1.5×10^{-3}	0.723	(0.011)	0.721

			PRS_{IV}	1.15	(1.07-1.24)	2.6×10^{-4}	0.723	(0.011)	0.722
Pancreas	493	22	PRS_{β}	1.49	(1.36-1.62)	1.3×10^{-18}	0.742	(0.012)	0.745
			PRS_{unw}	1.44	(1.31-1.57)	1.1×10^{-15}	0.738	(0.012)	0.741
			PRS_{IV}	1.49	(1.37-1.63)	5.2×10^{-19}	0.743	(0.012)	0.745
Oral cavity/ pharynx	481	14	PRS_{β}	1.11	(1.01-1.21)	2.3×10^{-2}	0.686	(0.015)	0.702
			PRS_{unw}	1.11	(1.02-1.22)	1.9×10^{-2}	0.686	(0.015)	0.702
			PRS_{IV}	1.12	(1.02-1.23)	1.3×10^{-2}	0.687	(0.015)	0.702
Lymphocytic leukemia	340	75	PRS_{β}	1.45	(1.31-1.61)	8.0×10^{-13}	0.735	(0.016)	0.719
			PRS_{unw}	1.67	(1.51-1.86)	1.2×10^{-21}	0.755	(0.015)	0.736
			PRS_{IV}	1.70	(1.53-1.88)	6.3×10^{-23}	0.756	(0.015)	0.738
Thyroid	191	12	PRS_{β}	1.57	(1.36-1.82)	5.7×10^{-10}	0.666	(0.023)	0.679
			PRS_{unw}	1.55	(1.34-1.78)	1.9×10^{-9}	0.671	(0.023)	0.676
			PRS_{IV}	1.75	(1.53-2.01)	1.9×10^{-15}	0.692	(0.022)	0.701

1. Hazard ratio estimates are adjusted for age at assessment (years), sex (if applicable), family history of cancer (for sites with available self-reported information), genotyping array, the first 15 genetic ancestry principal components, and any additional risk factors applicable to each cancer listed in Supplementary Table 1
2. Harrell's C-index was calculated as a weighted average between 1 and 5 years of follow-up
3. AUC values were estimated at 5 years of follow-up

Supplementary Table 5: Assessment of model discrimination for each cancer comparing different combinations of conventional risk factors and polygenic risk scores (PRS).

Cancer Site	Cases	Model specification	C index[2]	(C SE)	AUC[3]	Pseudo R^2
Prostate	4740	Age	0.710	(0.004)	0.713	0.349
		Age + family history	0.716	(0.004)	0.720	0.366
		Age + PRS_{IV} (instead of family history)	0.763	(0.004)	0.766	0.496
		Age + family history + PRS_{IV}	0.768	(0.004)	0.769	0.510
Testis	52	Age	0.627	(0.045)	0.658	0.184
		Age + PRS_{IV}	0.766	(0.033)	0.787	0.605
Breast	4760	Age	0.543	(0.005)	0.548	0.017
		Age + family history	0.559	(0.005)	0.562	0.031
		Age + PRS_{IV} (instead of family history)	0.620	(0.005)	0.626	0.122
		Age + family history + other predictors	0.572	(0.005)	0.573	0.043
		Age + family history + other predictors + PRS_{IV}	0.635	(0.004)	0.637	0.146
Endometrium	643	Age	0.631	(0.012)	0.631	0.127
		Age + family history	0.629	(0.012)	0.632	0.129
		Age + family history + other predictors	0.744	(0.011)	0.747	0.463
		Age + family history + other predictors + PRS_{IV}	0.749	(0.011)	0.754	0.486
Ovary	445	Age	0.607	(0.016)	0.620	0.106
		Age + family history	0.611	(0.016)	0.622	0.111
		Age + family history + other predictors	0.641	(0.015)	0.643	0.151
		Age + family history + other predictors + PRS_{IV}	0.654	(0.015)	0.660	0.193
Cervix[4]	282	Age	0.729	(0.017)	0.719	0.346
		Age + other predictors	0.736	(0.018)	0.731	0.386
		Age + other predictors + PRS_{IV}	0.749	(0.017)	0.745	0.437
Colon/rectum	2725	Age + sex	0.678	(0.006)	0.680	0.235
		Age + sex + family history	0.679	(0.006)	0.681	0.239
		Age + sex + PRS (instead of family history)	0.708	(0.006)	0.708	0.319
		Age + sex + family history + other predictors	0.686	(0.006)	0.688	0.258
		Age + sex + family history + other predictors + PRS_{IV}	0.716	(0.006)	0.716	0.345
Melanoma	1805	Age + sex	0.597	(0.008)	0.592	0.063
		Age + sex + other predictors	0.622	(0.008)	0.616	0.100
		Age + sex + other predictors + PRS_{IV}	0.664	(0.008)	0.654	0.180
Lung	1541	Age + sex	0.706	(0.007)	0.704	0.307
		Age + sex + family history	0.713	(0.007)	0.714	0.333
		Age + sex + PRS (instead of family history)	0.711	(0.007)	0.710	0.322
		Age + sex + family history + other predictors	0.846	(0.006)	0.843	0.789
		Age + sex + family history + other predictors + PRS_{IV}	0.849	(0.006)	0.846	0.799
Never smokers	207	Age + sex + family history + other predictors	0.709	(0.020)	0.712	0.320
		Age + sex + family history + other predictors + PRS_{IV}	0.723	(0.020)	0.723	0.354
Smokers	1334	Age + sex + family history + other predictors	0.805	(0.007)	0.804	0.641

		Age + sex + family history + other predictors + PRS_{IV}	0.809	(0.007)	0.808	0.657
NHL	970	Age + sex	0.667	(0.010)	0.669	0.207
		Age + sex + PRS_{IV}	0.674	(0.010)	0.677	0.227
Bladder	890	Age + sex	0.792	(0.008)	0.784	0.548
		Age + sex + other predictors	0.808	(0.008)	0.796	0.595
		Age + sex + other predictors + PRS_{IV}	0.814	(0.008)	0.804	0.628
Kidney	612	Age + sex	0.685	(0.012)	0.687	0.253
		Age + sex + other predictors	0.716	(0.011)	0.713	0.338
		Age + sex + other predictors + PRS_{IV}	0.723	(0.011)	0.722	0.366
Pancreas	493	Age + sex	0.692	(0.014)	0.695	0.273
		Age + sex + family history + other predictors	0.714	(0.013)	0.715	0.336
		Age + sex + family history + other predictors + PRS_{IV}	0.743	(0.012)	0.745	0.439
Oral cavity / pharynx	481	Age + sex	0.610	(0.014)	0.627	0.117
		Age + sex + other predictors	0.681	(0.015)	0.693	0.332
		Age + sex + other predictors + PRS_{IV}	0.687	(0.015)	0.702	0.356
Lymphocytic leukemia	340	Age + sex	0.695	(0.016)	0.688	0.255
		Age + sex + PRS_{IV}	0.756	(0.015)	0.738	0.415
Thyroid	191	Age + sex	0.577	(0.024)	0.590	0.060
		Age + sex + other predictors	0.592	(0.024)	0.604	0.079
		Age + sex + other predictors + PRS_{IV}	0.692	(0.022)	0.701	0.310

1. Harrell's C-index was calculated as a weighted average between 1 and 5 years of follow-up
2. AUC values were estimated at 5 years of follow-up
3. R^2 values correspond to the measure of explained variation by Royston P (2006) [Reference 10]
4. Incorporating time-varying PRS effects did not have an impact on predictive performance (AUC=0.745). As an additional sensitivity analysis we estimated the AUC using scaled Schoenfeld residuals, to account for non-proportionality in the PRS effects, which yielded AUC=0.772.

Supplementary Table 6: Percentile net reclassification improvement (NRI) index comparing the most comprehensive conventional risk factor model for each cancer with the model that incorporates the polygenic risk score (PRS) in addition to these risk factors. Event NRI (NRI_e) and non-event NRI (NRI_{ne}) quantify reclassification improvement in cases and event-free individuals, respectively. Bootstrapped confidence intervals were obtained based on 1000 replicates.

Cancer Site	NRI[1]	(95% CI)	NRI_e[2]	(95% CI)	NRI_{ne}[3]	(95% CI)
Prostate	0.444	(0.420, 0.467)	0.441	(0.417, 0.463)	0.003	(-0.0002, 0.007)
Testis	0.368	(0.174, 0.538)	0.385	(0.192, 0.555)	-0.018	(-0.021, -0.014)
Breast	0.384	(0.365, 0.403)	0.370	(0.351, 0.388)	0.014	(0.010, 0.018)
Endometrium	0.058	(0.019, 0.098)	0.060	(0.021, 0.099)	-0.002	(-0.004, 0.0003)
Ovary	0.173	(0.097, 0.236)	0.168	(0.093, 0.233)	0.005	(0.001, 0.008)
Cervix	0.073	(-0.001, 0.152)	0.075	(0.001, 0.156)	-0.002	(-0.005, 0.001)
Colon/rectum	0.273	(0.244, 0.301)	0.271	(0.242, 0.298)	0.003	(0.0003, 0.005)
Melanoma	0.293	(0.260, 0.323)	0.285	(0.250, 0.315)	0.008	(0.005, 0.011)
Lung	0.044	(0.020, 0.070)	0.029	(0.004, 0.055)	0.015	(0.013, 0.017)
NHL	0.119	(0.078, 0.161)	0.116	(0.074, 0.157)	0.004	(0.002, 0.006)
Bladder	0.116	(0.069, 0.164)	0.116	(0.069, 0.164)	0.0003	(-0.002, 0.002)
Kidney	0.065	(0.026, 0.103)	0.069	(0.030, 0.107)	-0.004	(-0.006, -0.003)
Pancreas	0.228	(0.174, 0.284)	0.228	(0.173, 0.284)	0.001	(-0.002, 0.003)
Oral cavity / pharynx	0.065	(0.022, 0.107)	0.069	(0.026. 0.111)	-0.004	(-0.006, -0.002)
Lymphocytic leukemia	0.307	(0.227, 0.388)	0.312	(0.232, 0.394)	-0.004	(-0.007, -0.002)
Thyroid	0.415	(0.330, 0.507)	0.409	(0.324, 0.501)	0.006	(0.003, 0.008)

1. $NRI = NRI_e + NRI_{ne}$
2. Difference in proportions of subjects with events (incident cancer) correctly reclassified to a higher-risk category minus those reclassified to a lower-risk category
3. Difference in proportions of subjects without events (cancer-free) correctly reclassified to a lower-risk category minus those reclassified to a higher-risk category

Supplementary Table 7: P-values for differences in mean absolute risk between strata based on genetic and other risk factor profiles depicted in Figures 3, 4, and 5. Low polygenic risk score (PRS) corresponds to ≤20th percentile, average PRS is defined as >20th to <80th percentile, and high PRS includes individuals in the ≥80th percentile of the normalized PRS distribution. Individuals below the median of the modifiable risk factor distribution were classified as having reduced risk, whereas those above the median had elevated risk. Family history was based on self-reported cancers in first-degree relatives. All p-values are based on a two-sample t-test calculated for differences in risk at age 60 except for pre-menopausal breast cancer (mean risk at age 50).

Contrast	Cancer Site							
	Prostate	Breast	Colon/rectal	Lung				
Low PRS / Family history vs. Low PRS / No family history	6.5×10^{-37}	1.4×10^{-62}	9.7×10^{-26}	2.2×10^{-11}				
Low PRS / Family history vs. Average PRS / No family history	4.5×10^{-25}	4.6×10^{-32}	3.9×10^{-60}	1.6×10^{-7}				
Average PRS / No family history vs. Average PRS / Family history	4.1×10^{-119}	4.0×10^{-148}	4.7×10^{-62}	6.8×10^{-26}				
Average PRS / Family history vs. High PRS / No family history	5.9×10^{-66}	1.0×10^{-78}	1.0×10^{-194}	1.5×10^{-7}				
High PRS / No family history vs. High PRS / Family history	4.4×10^{-31}	8.7×10^{-49}	2.0×10^{-21}	4.3×10^{-13}				
Low PRS / Family history vs. High PRS / No family history	-	-	-	0.031				
Low PRS / No family history vs. Average PRS / No family history	-	-	-	4.6×10^{-10}				
Average PRS / No family history vs. High PRS / No family history	-	-	-	3.8×10^{-16}				
	Breast: Pre-menopausal	Breast: Post-menopausal	Colon/rectal	Melanoma	Lung	Bladder	Kidney	Oral/ pharynx
Low PRS / Reduced modifiable vs. Low PRS / Elevated modifiable	7.9×10^{-20}	3.5×10^{-69}	$<10^{-324}$	1.4×10^{-269}	4.1×10^{-192}	1.3×10^{-199}	1.2×10^{-318}	2.8×10^{-209}
Low PRS / Elevated modifiable vs. Average PRS / Reduced modifiable	9.1×10^{-59}	1.2×10^{-288}	1.8×10^{-42}	1.3×10^{-165}	1.7×10^{-181}	7.9×10^{-78}	1.2×10^{-224}	6.0×10^{-173}
Average PRS / Reduced modifiable vs. Average PRS / Elevated modifiable	1.5×10^{-36}	6.3×10^{-165}	$<10^{-324}$	$<10^{-324}$	$<10^{-324}$	$<10^{-324}$	$<10^{-324}$	$<10^{-324}$
Average PRS / Elevated modifiable vs. High PRS / Reduced modifiable	2.4×10^{-77}	1.8×10^{-122}	7.8×10^{-49}	3.5×10^{-139}	$<10^{-324}$	$<10^{-324}$	$<10^{-324}$	$<10^{-324}$
High PRS / Reduced modifiable vs. High PRS / Elevated modifiable	3.4×10^{-14}	1.7×10^{-40}	3.6×10^{-301}	3.3×10^{-211}	1.9×10^{-181}	2.2×10^{-179}	1.9×10^{-323}	2.3×10^{-203}
Low PRS / Elevated modifiable vs. Average PRS / Elevated modifiable	-	-	-	-	1.1×10^{-13}	5.3×10^{-100}	3.6×10^{-56}	1.1×10^{-12}
Average PRS / Elevated modifiable vs. High PRS / Elevated modifiable	-	-	-	-	1.6×10^{-19}	5.6×10^{-77}	1.7×10^{-52}	1.6×10^{-13}
Low PRS / Elevated modifiable vs. High PRS / Reduced modifiable	-	-	-	-	2.7×10^{-161}	0.99	3.6×10^{-98}	7.3×10^{-132}
Low PRS / Reduced modifiable vs. Average PRS / Reduced modifiable	-	-	-	-	7.7×10^{-128}	1.6×10^{-133}	4.8×10^{-88}	3.4×10^{-88}
Average PRS / Reduced modifiable vs. High PRS / Reduced modifiable	-	-	-	-	1.4×10^{-208}	3.1×10^{-79}	5.2×10^{-67}	2.5×10^{-65}

Supplementary Table 8: Assessment of interaction on the absolute risk scale between ordinal polygenic risk score (PRS) categories (average: 20th to <80th percentile; high: ≥80th percentile vs. low: ≤20th percentile) and family history of cancer (yes vs. none) or elevated modifiable risk factor profile (summary score >50th percentile vs. ≤50th percentile). Coefficients and p-values for interaction terms were estimated using linear regression models with the predicted absolute risk of cancer at age 60 (age 50 for pre-menopausal breast cancer) as the outcome.

Cancer Site	Interaction Combination	Interaction		Overall Interaction P-value[1]
		Coefficient	P-value	
Prostate	PRS(average) * family history(yes)	7.8×10^{-3}	7.0×10^{-15}	9.0×10^{-128}
	PRS(high) * family history(yes)	0.026	7.4×10^{-104}	
Breast	PRS(average) * family history(yes)	3.0×10^{-3}	2.9×10^{-9}	1.2×10^{-98}
	PRS(high) * family history(yes)	0.011	6.4×10^{-80}	
Colorectal	PRS(average) * family history(yes)	3.3×10^{-4}	0.030	8.7×10^{-14}
	PRS(high) * family history(yes)	1.2×10^{-3}	2.8×10^{-12}	
Lung	PRS(average) * family history(yes)	-5.4×10^{-5}	0.88	7.0×10^{-4}
	PRS(high) * family history(yes)	1.1×10^{-3}	5.9×10^{-3}	
Breast (Pre-menopausal)	PRS(average) * modifiable(elevated)	1.3×10^{-3}	0.021	4.9×10^{-7}
	PRS(high) * modifiable(elevated)	3.6×10^{-3}	1.7×10^{-7}	
Breast (Post-menopausal)	PRS(average) * modifiable(elevated)	1.7×10^{-3}	4.8×10^{-6}	6.9×10^{-24}
	PRS(high) * modifiable(elevated)	4.6×10^{-3}	6.5×10^{-24}	
Endometrium	PRS(average) * modifiable(elevated)	5.0×10^{-4}	4.0×10^{-5}	5.3×10^{-16}
	PRS(high) * modifiable(elevated)	1.2×10^{-3}	1.2×10^{-16}	
Ovary	PRS(average) * modifiable(elevated)	1.5×10^{-4}	5.9×10^{-7}	1.4×10^{-19}
	PRS(high) * modifiable(elevated)	3.5×10^{-4}	1.9×10^{-20}	
Cervix	PRS(average) * modifiable(elevated)	7.7×10^{-5}	0.072	9.1×10^{-5}
	PRS(high) * modifiable(elevated)	2.2×10^{-4}	2.6×10^{-5}	
Colorectum	PRS(average) * modifiable(elevated)	1.0×10^{-3}	3.0×10^{-38}	1.3×10^{-208}
	PRS(high) * modifiable(elevated)	2.9×10^{-3}	1.3×10^{-194}	
Melanoma	PRS(average) * modifiable(elevated)	5.1×10^{-4}	3.8×10^{-28}	3.3×10^{-122}
	PRS(high) * modifiable(elevated)	1.3×10^{-3}	3.9×10^{-118}	
Lung	PRS(average) * modifiable(elevated)	1.2×10^{-3}	3.6×10^{-8}	1.1×10^{-37}
	PRS(high) * modifiable(elevated)	3.3×10^{-3}	4.3×10^{-37}	
Bladder	PRS(average) * modifiable(elevated)	3.4×10^{-4}	3.3×10^{-11}	1.5×10^{-50}
	PRS(high) * modifiable(elevated)	9.3×10^{-4}	1.2×10^{-49}	
Kidney	PRS(average) * modifiable(elevated)	1.8×10^{-4}	9.7×10^{-9}	5.5×10^{-29}
	PRS(high) * modifiable(elevated)	4.2×10^{-4}	1.6×10^{-29}	
Pancreas	PRS(average) * modifiable(elevated)	1.6×10^{-4}	3.7×10^{14}	2.1×10^{-91}
	PRS(high) * modifiable(elevated)	5.2×10^{-4}	6.5×10^{-85}	
Oral cavity/pharynx	PRS(average) * modifiable(elevated)	1.1×10^{-4}	9.6×10^{-4}	5.2×10^{-11}
	PRS(high) * modifiable(elevated)	2.7×10^{-4}	1.1×10^{-11}	
Thyroid	PRS(average) * modifiable(elevated)	4.7×10^{-5}	2.1×10^{-4}	5.4×10^{-30}
	PRS(high) * modifiable(elevated)	1.7×10^{-4}	6.6×10^{-28}	

1. Calculated based on a chi-square test with 2 degrees of freedom

Supplementary Table 9: Population attributable fractions (PAF) were estimated at 5 years of follow-up time for the top 20% ($\geq 80^{th}$ percentile) of the modifiable risk factor and polygenic risk score (PRS) distributions, respectively, and family history of cancer at the relevant site. PAF estimates and corresponding p-values were derived from Cox proportional hazard regression models that were adjusted for age at enrollment, sex, family history of cancer (if available), genotyping array, and the top 15 genetic ancestry principal components.

	High Genetic Risk			High Modifiable Risk			Family History		
	PAF	(95% CI)	P-value	PAF	(95% CI)	P-value	PAF	(95% CI)	P-value
Prostate	0.232	(0.215 - 0.249)	5.5×10^{-158}	-	-	-	0.055	(0.045 - 0.065)	1.4×10^{-25}
Testis	0.303	(0.135 - 0.472)	4.5×10^{-4}	-	-	-	-	-	-
Breast	0.168	(0.151 - 0.184)	4.9×10^{-87}	0.037	(0.019 - 0.054)	6.4×10^{-5}	0.051	(0.040 - 0.063)	3.3×10^{-18}
Pre-menopausal	0.173	(0.136 - 0.210)	3.1×10^{-20}	0.004	(-0.010 - 0.018)	0.54	0.044	(0.021 - 0.068)	2.5×10^{-4}
Post-menopausal	0.159	(0.139 - 0.179)	$4.\times10^{-54}$	0.044	(0.019 - 0.071)	6.9×10^{-4}	0.053	(0.039 - 0.068)	2.5×10^{-13}
Endometrium	0.043	(0.002 - 0.084)	0.039	0.353	(0.303 - 0.404)	1.5×10^{-43}	0.042	(-0.020 - 0.103)	0.18
Ovary[1]	0.082	(0.031 - 0.134)	1.6×10^{-3}	0.100	(0.038 - 0.161)	1.4×10^{-3}	0.025	(-0.012 - 0.063)	0.19
Cervix	0.123	(0.057 - 0.190)	2.7×10^{-4}	0.065	(-0.001 - 0.130)	0.053	-	-	-
Colon/rectum	0.167	(0.145 - 0.190)	9.2×10^{-50}	0.111	(0.085 - 0.136)	1.6×10^{-17}	0.027	(0.012 - 0.042)	5.3×10^{-4}
Melanoma	0.139	(0.112 - 0.166)	1.3×10^{-23}	0.066	(0.039 - 0.093)	1.1×10^{-6}	-	-	-
Lung	0.040	(0.013 - 0.066)	3.1×10^{-3}	0.636	(0.606 - 0.666)	1.4×10^{-376}	0.089	(0.065 - 0.114)	1.0×10^{-12}
Never smokers[2]	0.077	(0.002 - 0.151)	0.045	0.049	(-0.076 - 0.174)	0.44	-0.013	(-0.066 - 0.039)	0.62
Smokers	0.035	(0.007 - 0.063)	0.015	0.663	(0.620 - 0.706)	3.2×10^{-200}	0.105	(0.078 - 0.132)	2.8×10^{-14}
NHL	0.053	(0.020 - 0.087)	1.9×10^{-3}	-	-	-	-	-	-
Bladder	0.085	(0.048 - 0.121)	4.7×10^{-6}	0.189	(0.140 - 0.237)	4.1×10^{-14}	-	-	-
Kidney	0.046	(0.005 - 0.087)	0.026	0.210	(0.160 - 0.260)	2.4×10^{-16}	-	-	-
Pancreas[3]	0.133	(0.082 - 0.184)	2.9×10^{-7}	0.118	(0.064 - 0.172)	1.9×10^{-5}	0.134	(0.063 - 0.205)	2.3×10^{-4}
Oral cavity / pharynx	0.006	(-0.038 - 0.051)	0.78	0.310	(0.253 - 0.368)	4.0×10^{-26}	-	-	-
Lymphocytic leukemia	0.269	(0.204 - 0.334)	7.4×10^{-16}	-	-	-	-	-	-
Thyroid	0.268	(0.180 - 0.355)	1.7×10^{-9}	0.202	(0.039 - 0.366)	0.015	-	-	-

1. Family history variable refers to self-reported breast cancer in a first-degree relative
2. The only modifiable risk factor is air pollution, modeled here as a categorical variable corresponding to $PM_{2.5}$ levels above the median
3. Family history variable refers to self-reported breast, prostate, lung, or bowel cancer in a first-degree relative

Supplementary Figure 1: Calibration plots comparing predicted and observed event probabilities for each of the 16 cancers examined. The most comprehensive risk factor model available is plotted in black and the same model with the addition of the polygenic risk score (PRS) is overlaid in red. Goodness of fit p-values (P_{GoF}) are based on the Hosmer & Lemeshow test.

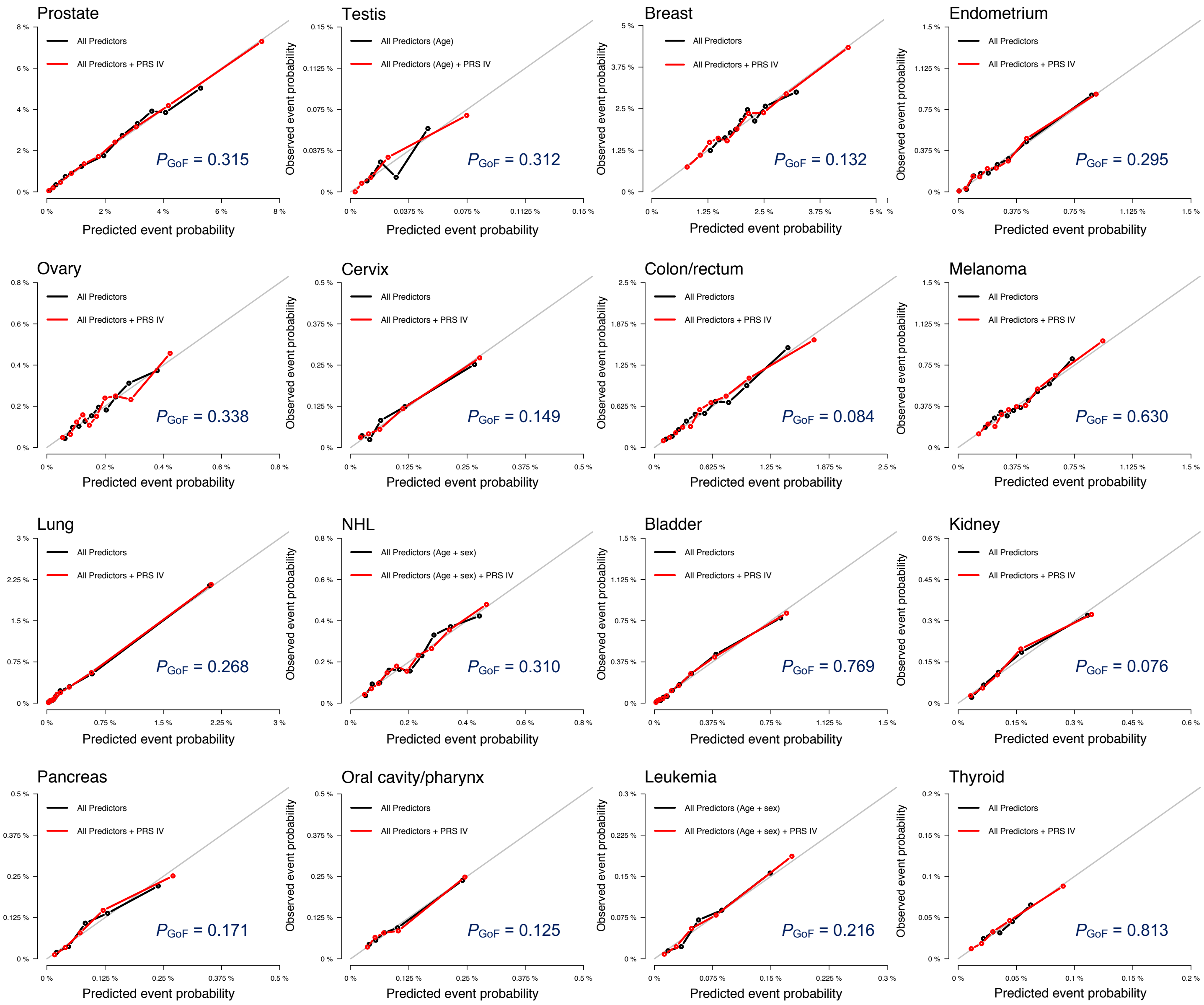

Supplementary Figure 2: Plots of Schoenfeld residuals and corresponding p-values for the cervical cancer polygenic risk score (PRS_{IV}). Below is a comparison of PRS_{IV} effects estimated using a time-varying model (blue) and hazard ratio estimated under the proportionality assumption (red).

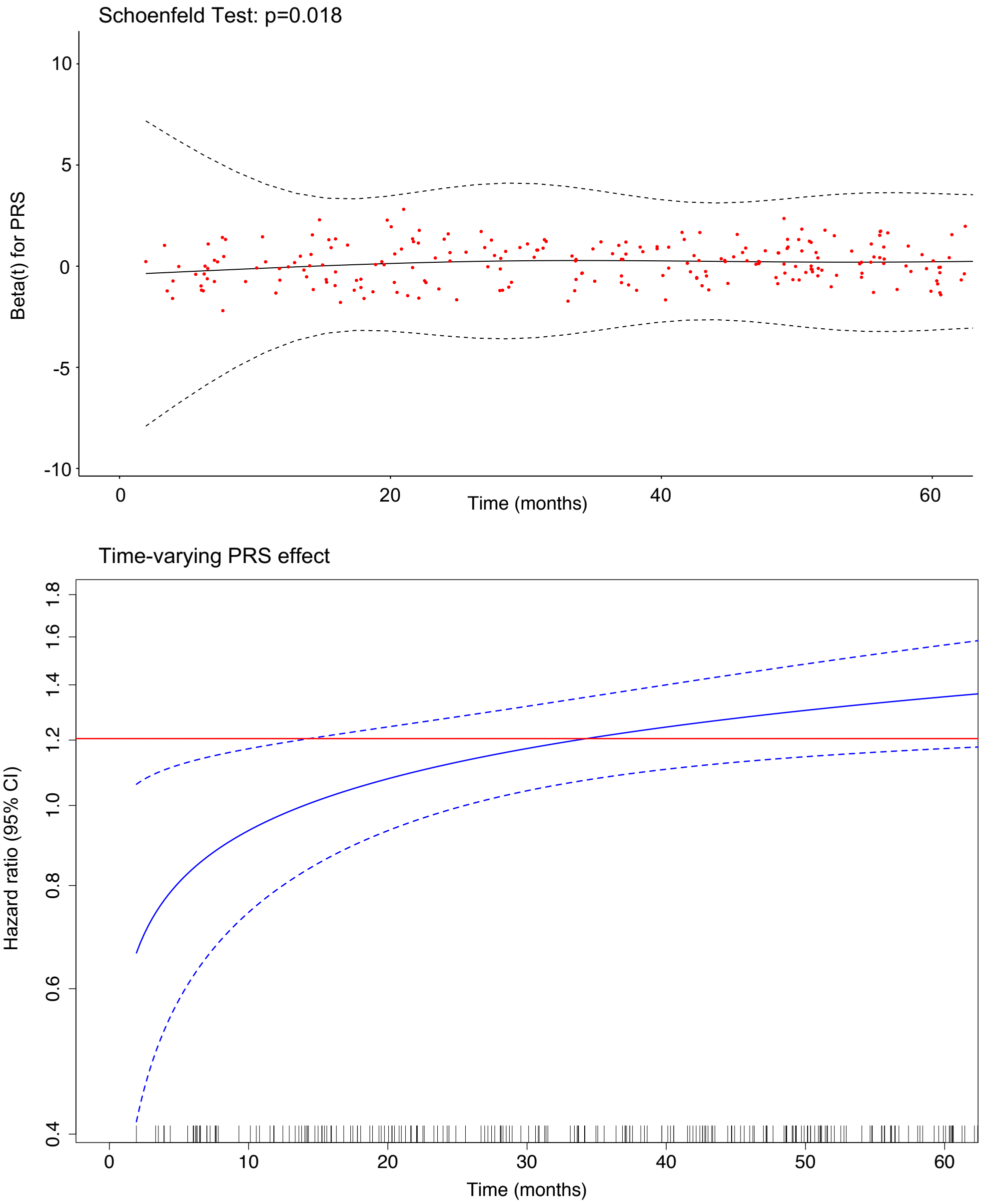

Supplementary Figure 3: Predicted 5-year absolute risks across strata defined by PRS and modifiable risk factors, where applicable, and age categories. Low genetic risk is based on percentiles of the standardized polygenic risk score (PRS). Low PRS corresponds to ≤20th percentile, average PRS is defined as >20th to <80th percentile, and high PRS includes individuals in the ≥80th percentile. Individuals below the median of the modifiable risk factor distribution were considered to have reduced risk, whereas those above the median had elevated risk. Absolute risks are visualized as box plots corresponding to the interquartile range (IQR), with outlying data points beyond 1.5*IQR plotted individually.

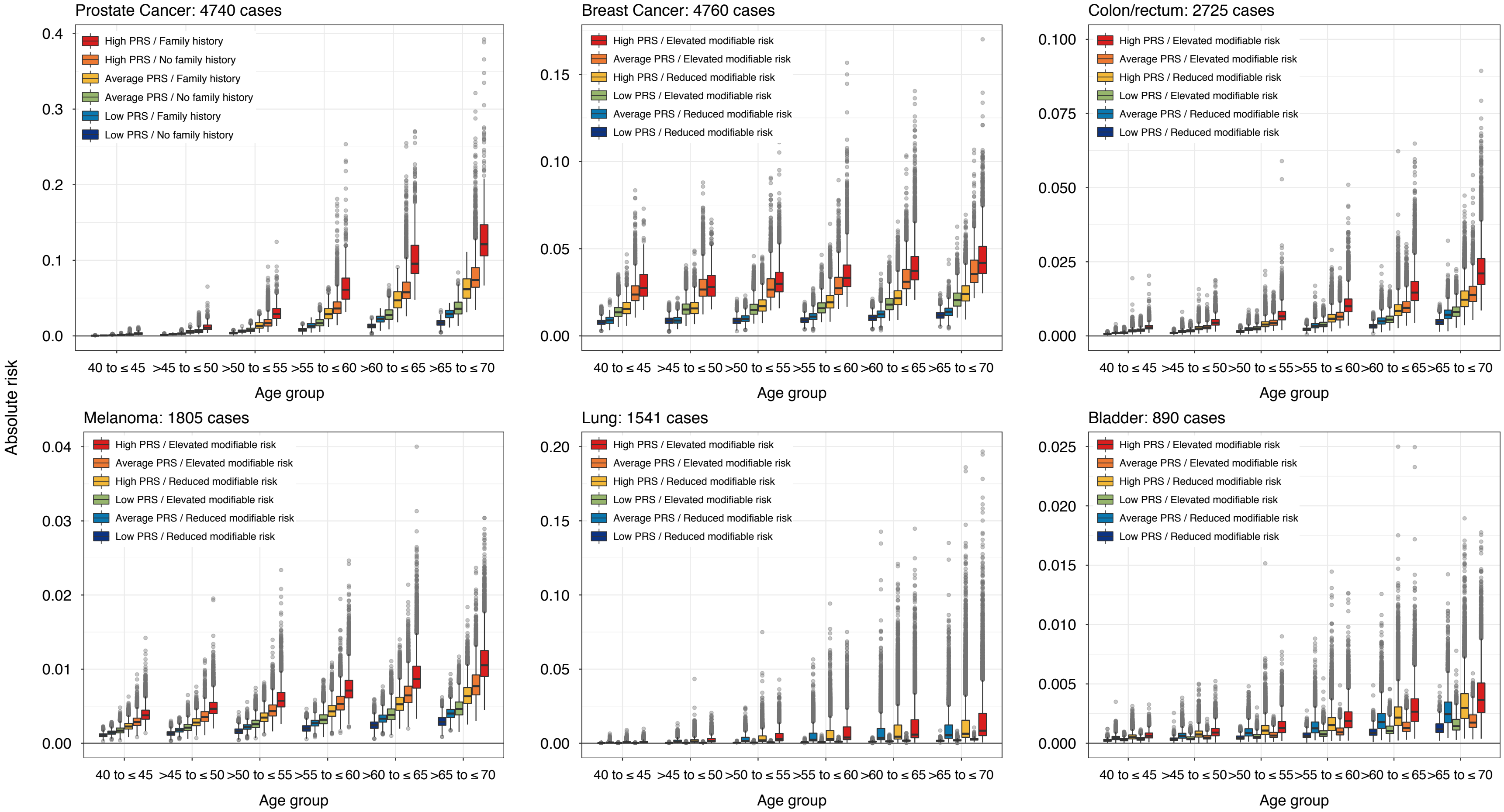

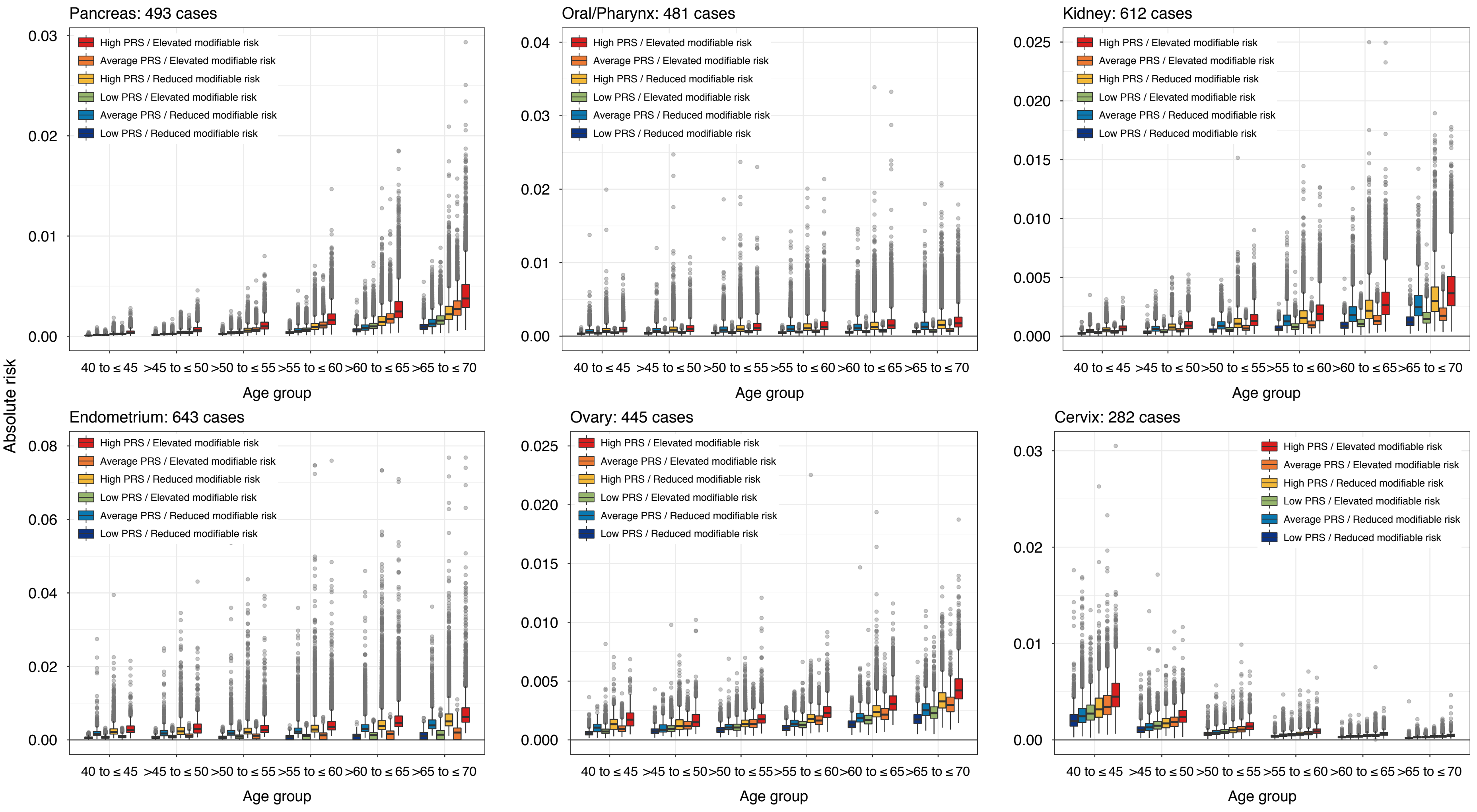
Pancreas: 493 cases
Oral/Pharynx: 481 cases
Kidney: 612 cases
Endometrium: 643 cases
Ovary: 445 cases
Cervix: 282 cases
High PRS / Elevated modifiable risk
Average PRS / Elevated modifiable risk
High PRS / Reduced modifiable risk
Low PRS / Elevated modifiable risk
Average PRS / Reduced modifiable risk
Low PRS / Reduced modifiable risk
Absolute risk
Age group
40 to ≤ 45
>45 to ≤ 50
>50 to ≤ 55
>55 to ≤ 60
>60 to ≤ 65
>65 to ≤ 70

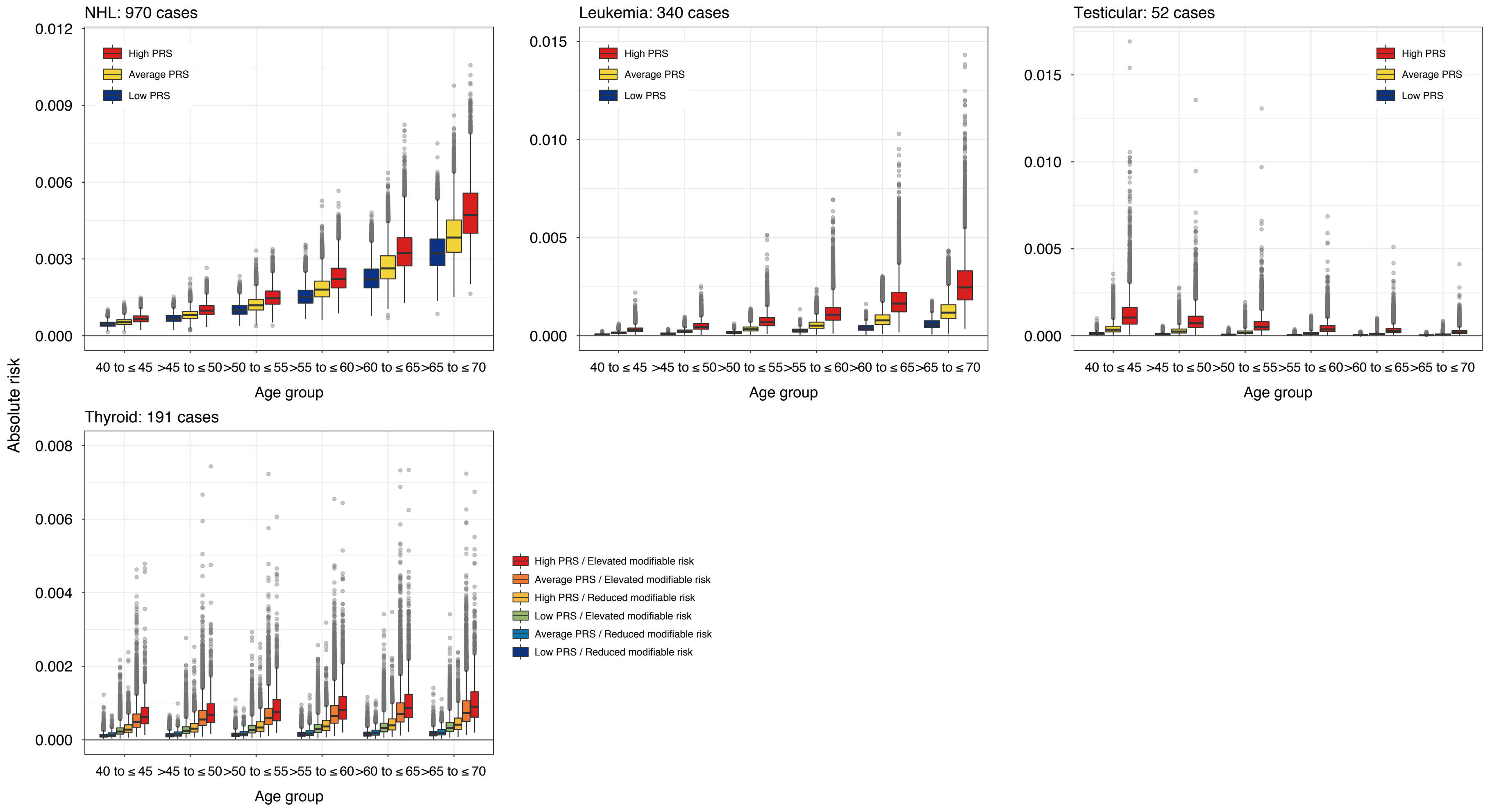

NHL: 970 cases
Leukemia: 340 cases
Testicular: 52 cases
Thyroid: 191 cases
Absolute risk
Age group
40 to ≤ 45
>45 to ≤ 50
>50 to ≤ 55
>55 to ≤ 60
>60 to ≤ 65
>65 to ≤ 70
High PRS
Average PRS
Low PRS
High PRS / Elevated modifiable risk
Average PRS / Elevated modifiable risk
High PRS / Reduced modifiable risk
Low PRS / Elevated modifiable risk
Average PRS / Reduced modifiable risk
Low PRS / Reduced modifiable risk

Supplementary Figure 4: Predicted 5-year absolute risk trajectories across strata defined by PRS and modifiable risk factors, where applicable. Low genetic risk is based on percentiles of the standardized polygenic risk score (PRS). Low PRS corresponds to ≤20th percentile, average PRS is defined as >20th to <80th percentile, and high PRS includes individuals in the ≥80th percentile. Individuals below the median of the modifiable risk factor distribution were considered to have reduced risk, whereas those above the median had elevated risk. P-values are based on t-tests comparing mean absolute risk in each stratum at age 60 or age 50 for cervical and testicular cancers. All statistical tests were two-sided.

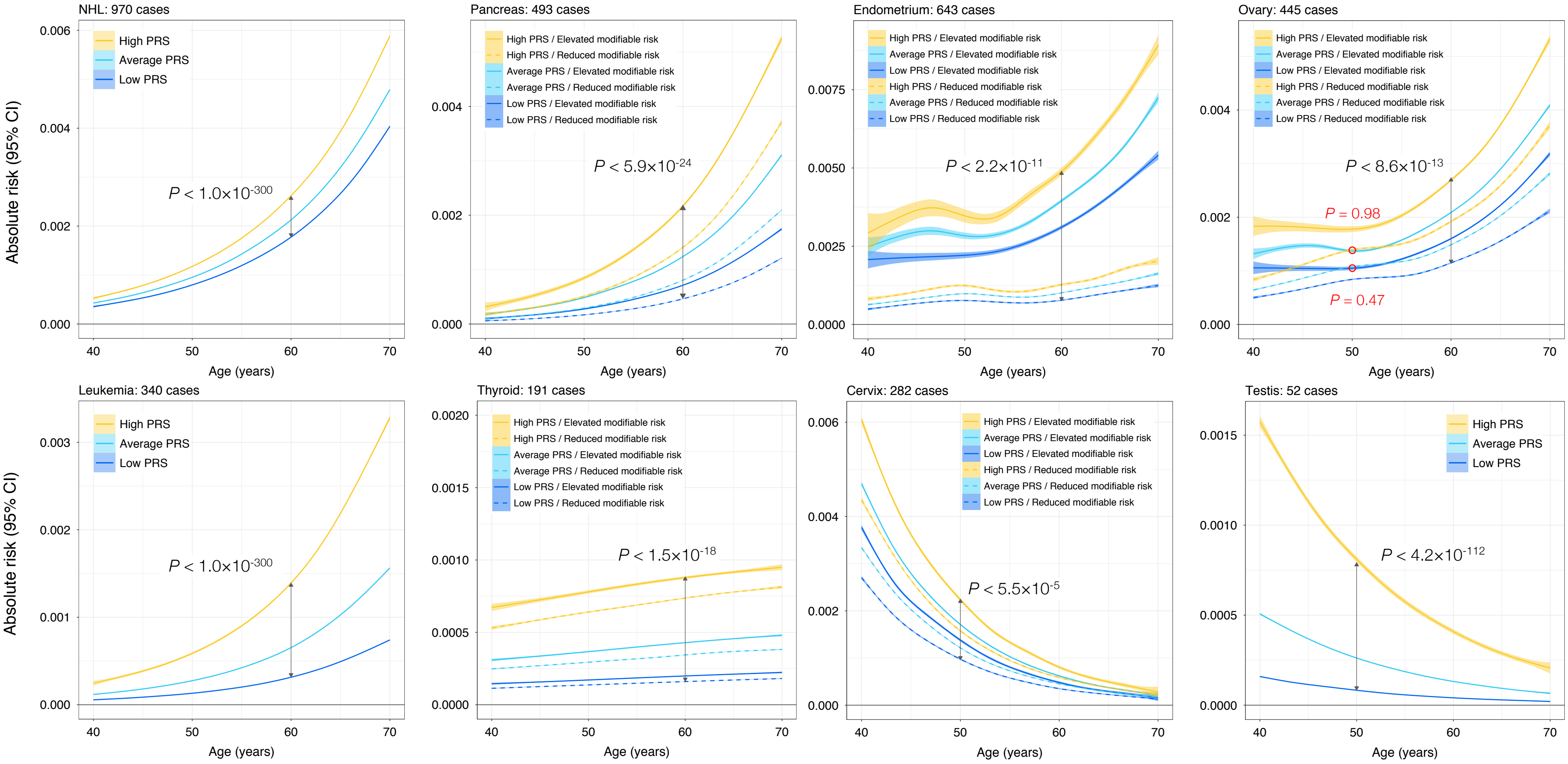

Note: for cervical cancer estimates of absolute risk were derived from the Cox proportional hazards model without time-varying PRS effects
