# Supplementary material for: Pan-cancer analysis demonstrates that integrating polygenic risk scores with modifiable risk factors improves risk prediction
Source: Nat Commun. 2020 Nov 27;11:6084. doi: 10.1038/s41467-020-19600-4 (PMC7695829; doi:10.1038/s41467-020-19600-4)
Supplement: Supplementary file 2 — Description of Additional Supplementary Files [file 41467_2020_19600_MOESM2_ESM.pdf]

Description of Additional Supplementary Files

File Name: Supplementary Data 1

Description: Genetic variants included in each cancer-specific polygenic risk score with risk alleles and corresponding weights.
