# Supplementary material for: Pan-cancer analysis demonstrates that integrating polygenic risk scores with modifiable risk factors improves risk prediction
Source: Nat Commun. 2020 Nov 27;11:6084. doi: 10.1038/s41467-020-19600-4 (PMC7695829; doi:10.1038/s41467-020-19600-4)
Supplement: Supplementary file 4 — Reporting Summary [file 41467_2020_19600_MOESM4_ESM.pdf]

nature research

Corresponding author(s): John S. Witte
Mattias Johansson

Last updated by author(s): Sep 11, 2020

Reporting Summary

Nature Research wishes to improve the reproducibility of the work that we publish. This form provides structure for consistency and transparency in reporting. For further information on Nature Research policies, see Authors & Referees and the Editorial Policy Checklist.

Statistics

For all statistical analyses, confirm that the following items are present in the figure legend, table legend, main text, or Methods section.

n/a	Confirmed	
☐	☒	The exact sample size (*n*) for each experimental group/condition, given as a discrete number and unit of measurement
☐	☒	A statement on whether measurements were taken from distinct samples or whether the same sample was measured repeatedly
☐	☒	The statistical test(s) used AND whether they are one- or two-sided<br>*Only common tests should be described solely by name; describe more complex techniques in the Methods section.*
☐	☒	A description of all covariates tested
☐	☒	A description of any assumptions or corrections, such as tests of normality and adjustment for multiple comparisons
☐	☒	A full description of the statistical parameters including central tendency (e.g. means) or other basic estimates (e.g. regression coefficient) AND variation (e.g. standard deviation) or associated estimates of uncertainty (e.g. confidence intervals)
☐	☒	For null hypothesis testing, the test statistic (e.g. F, t, r) with confidence intervals, effect sizes, degrees of freedom and P value noted<br>*Give P values as exact values whenever suitable.*
☒	☐	For Bayesian analysis, information on the choice of priors and Markov chain Monte Carlo settings
☒	☐	For hierarchical and complex designs, identification of the appropriate level for tests and full reporting of outcomes
☐	☒	Estimates of effect sizes (e.g. Cohen's *d*, Pearson's *r*), indicating how they were calculated

Our web collection on statistics for biologists contains articles on many of the points above.

Software and code

Policy information about availability of computer code

Data collection	No software was used
Data analysis	R package riskRegression (version: 2019.11.03)<br>R package survival (version: 3.1.8)<br>R package rms (version: 5.1.4)<br>R package survMisc (version 0.5.5)<br>R package risksetROC (version 1.0.4)<br>R package AF (version: 0.1.5)<br>R code for calculating percentile-based NRI was obtained from the supplemental files of the original publication (McKearnan SB et al. 2018; PMID: 29304237)<br>KING version 2.0 (http://people.virginia.edu/~wc9c/KING/)<br>Principal components were calculated by the UK Biobank team using fastPCA and provided with the genetic data

For manuscripts utilizing custom algorithms or software that are central to the research but not yet described in published literature, software must be made available to editors/reviewers. We strongly encourage code deposition in a community repository (e.g. GitHub). See the Nature Research guidelines for submitting code & software for further information.

Data

Policy information about availability of data

All manuscripts must include a data availability statement. This statement should provide the following information, where applicable:

- Accession codes, unique identifiers, or web links for publicly available datasets
- A list of figures that have associated raw data
- A description of any restrictions on data availability

The UK Biobank in an open access resource, available at https://www.ukbiobank.ac.uk/researchers/. This research was conducted with approved access to UK Biobank data under application number 14105.

Field-specific reporting

Please select the one below that is the best fit for your research. If you are not sure, read the appropriate sections before making your selection.

☒ Life sciences ☐ Behavioural & social sciences ☐ Ecological, evolutionary & environmental sciences

For a reference copy of the document with all sections, see nature.com/documents/nr-reporting-summary-flat.pdf

Life sciences study design

All studies must disclose on these points even when the disclosure is negative.

Sample size	The UK Biobank is a population-based prospective cohort of 502,611 individuals in the United Kingdom. Study participants were aged 40 to 69 at recruitment between 2006 and 2010, at which time all participants provided detailed information about lifestyle and health-related factors, provided biological samples, and completed a range of physical measures. After relevant exclusions (described below) the final sample size available for analysis included 413,753 individuals (of these 22,755 were incident cancer cases). The final sample size is the maximum achievable based on our predetermined study criteria. This is sufficient for the present analysis since our aim is not to discover novel genetic associations, but rather to characterize the performance of genetic risk scores constructed using external, publicly available data.
Data exclusions	Exclusion criteria were predetermined. Analyses in the UK Biobank were limited to individuals with self-reported European ancestry with concordant self-reported and genetic sex. To further minimize potential population stratification, we excluded individuals for whom either of the first two ancestry PCs fell outside five standard deviations of the mean of the population. Based on a subset of genotyped autosomal variants with minor allele frequency (MAF) ≥0.01 and genotype call rate ≥97%, we excluded samples with call rates <97% and/or heterozygosity more than five standard deviations from the mean of the population. With the same subset of SNPs, we used KING to estimate relatedness among the samples. We excluded one individual from each pair of first-degree relatives, first prioritizing on maximizing the number of cancer cases and then maximizing the total number of individuals in the analyses. We also removed UK Biobank participants who withdrew consent at a later date and no longer wish their data to be included in UK Biobank analyses.
Replication	The polygenic risk scores (PRS) assessed in this analysis have been developed from publicly available, previously published data. The goal of the present analysis is to establish the predictive performance of these known, previously replicated genetic susceptibility variants. Each PRS was replicated for the target cancer at p-value <0.05 (two-sided). Our supplementary data file provides a full list of genetic variants used to construct each cancer-specific PRS, with the corresponding weights, to facilitate future follow up of our results.
Randomization	The UK Biobank study is a prospective population-based observational cohort, with no randomization or intervention component. Association analyses of polygenic risk scores (PRS) and relevant end-points (incident cancer/death/censored) were conducted using Cox proportional hazards regression and adjusted for the following minimum set of covariates: age at cohort enrollment, sex, first 15 genetic ancestry PCs, and genotyping array.
Blinding	The UK Biobank study is a prospective population-based observational cohort, with no treatment or specific control groups. Ascertainment of cancer status (conducted via linkage to national cancer registries) and genotyping in the UK Biobank was performed independently from each other. The researchers who carried out the analysis for this manuscript had no influence on how genotyping or cancer phenotype assessment was performed in the UK Biobank.

Reporting for specific materials, systems and methods

We require information from authors about some types of materials, experimental systems and methods used in many studies. Here, indicate whether each material, system or method listed is relevant to your study. If you are not sure if a list item applies to your research, read the appropriate section before selecting a response.

Materials & experimental systems

n/a	Involved in the study	
☒	☐	Antibodies
☒	☐	Eukaryotic cell lines
☒	☐	Palaeontology
☒	☐	Animals and other organisms
☐	☒	Human research participants
☒	☐	Clinical data

Methods

n/a	Involved in the study	
☒	☐	ChIP-seq
☒	☐	Flow cytometry
☒	☐	MRI-based neuroimaging

Human research participants

Policy information about studies involving human research participants

Population characteristics	The UK Biobank is a population-based prospective cohort of 502,611 men and women in the United Kingdom. Study participants were aged 40 to 69 at recruitment between 2006 and 2010, at which time all participants provided detailed information about lifestyle and health-related factors, provided biological samples, and completed a range of physical measures. Analyses were based on data from individuals of predominantly European ancestry, based on self-report and genetic ancestry principal components (PC's), and those with concordant self-reported and genetic sex.
Recruitment	The UK Biobank is a population-based cohort of 500,000 participants recruited in the United Kingdom between 2006-2010. Approximately 9.2 million individuals aged 40-69 years who lived within 25 miles of one of 22 assessment centers in England, Wales, and Scotland were invited to enter to cohort, and 5.5% completed the baseline assessment. The UK Biobank is not representative of the general population across several sociodemographic, physical, lifestyle and health-related characteristics, with evidence of a "healthy volunteer" selection bias, details of which are published elsewhere (Fry et al, Am J Epidemiol 2017;186:1026-34. PMID 28641372).
Ethics oversight	The study was approved by the UK Biobank data access committee under application 14105 (PI: John S. Witte). Informed consent was obtained from all participants.

Note that full information on the approval of the study protocol must also be provided in the manuscript.
